# Prevalence of PI*Z and PI*S alleles of alpha-1-antitrypsin deficiency in Finland

**DOI:** 10.3402/ecrj.v2.28829

**Published:** 2015-09-24

**Authors:** Jan Häggblom, Kaisa Kettunen, Jussi Karjalainen, Markku Heliövaara, Pekka Jousilahti, Seppo Saarelainen

**Affiliations:** 1 Sastamala Region's Social and Health Services, Sastamala, Finland; 2 Institute for Molecular Medicine Finland FIMM, Technology Center, University of Helsinki, Helsinki, Finland; 3 Allergy Centre, Tampere University Hospital, Tampere, Finland; 4 Department of Health, National Institute of Health and Welfare, Helsinki, Finland; 5 Department of Respiratory Medicine, Tampere University Hospital, Tampere, Finland

**Keywords:** alpha-1-antitrypsin deficiency, Europe, Finland, genetic epidemiology, PI*Z, PI*S, PI*M (Malton), prevalence, SERPINA1

## Abstract

The prevalence of PI*Z and PI*S alleles of *SERPINA1* gene related to alpha-1-antitrypsin deficiency has previously been estimated to be lower in Finland than in the other countries of Northern Europe. The prevalence of PI*M (Malton) has not been studied in Finland before. We determined alpha-1-antitrypsin PI*Z and PI*S and PI*M (Malton) genotypes from a representative population sample. The number of subjects was 6,354 in the PI*S and PI*M (Malton) genotyping. PI*Z genotyping was performed in a subsample of 2,482 subjects. The allele frequencies were PI*Z 19.7/1,000 and PI*S 10.2/1,000. No PI*M (Malton) was found. The number of carriers of PI*Z and PI*S is significantly higher than previously estimated. The prevalences are in line with the findings in the neighboring countries.

Alpha-1-antitrypsin deficiency is an autosomal codominant inherited disorder (1). It is one of the most common hereditary disorders in Europe. Persons with MM phenotype have normal structure of the alpha-1-antitrypsin protein. Most pathology related to this disease is linked to the Z allele, and in clinical practice most of the patients have a ZZ phenotype. The remaining patients mostly have SZ, MZ, and, in a smaller amount, other rare deficiency or null phenotypes (2). Alpha1-antitrypsin deficiency results from point mutations in the *SERPINA1* gene that distorts the structure of the alpha-1-antitrypsin protein. This causes an accumulation of polymers of Z alpha1-antitrypsin within hepatocytes to form inclusion bodies that are associated with juvenile cirrhosis and hepatocellular carcinoma (3). The lack of circulating protein predisposes the Z alpha1-antitrypsin homozygote to emphysema (3). M (Malton), like the Z allele, is associated with hepatic and pulmonary disease. In some Mediterranean regions, M (Malton) allele is more prevalent than Z and S alleles and can be incorrectly diagnosed as PI*M by isoelectric focusing (4, 5).

The prevalence of PI*Z and PI*S alleles of alpha-1-antitrypsin deficiency was studied in Finland over three decades ago (6–8). Based on these studies, the frequencies of PI*Z and PI*S alleles has been estimated as PI*Z 6.6/1,000 and PI*S 7.3/1,000, respectively (expressed as the total number of Z and S alleles per 1,000 genes of all PI types) (9, 10). The populations in these studies, however, have been relatively small and affected by selection. Moreover, the prevalences of PI*Z and PI*S in the neighboring countries Sweden and Estonia have been shown to be 2–3 times higher than earlier reported in Finland.

Significant differences in Y haplotype variation have been reported between eastern and western regions of Finland, indicating that two separate founder populations provided a substantial contribution to the Finnish gene pool (11). This has raised a question whether there is also a significant east–west difference in alpha-1-antitrypsin deficiency in Finland, since the prevalence of the Z allele has been estimated to be very low in the northeastern parts of Finland (8). The aim of our present paper was to determinate the prevalences of PI*Z, PI*S, and PI*M (Malton) in a representative sample of Finnish population and to analyze whether there is a significant east–west difference.

## Materials and methods

In Health 2000 Survey, conducted in Finland in 2000–2001, a nationally representative sample of 8,028 persons aged 30 years or over was drawn from the population register and invited to take part in a two-phased study. The first part included a postal questionnaire asking for information on lifestyle, health status, and chronic illness diagnosed by a physician. The second part included interviews by specially trained nurses and several physical measurements (including a blood sample). In the first part, 99% responded to the questionnaire (7,979/8,028). Of these 7,087 (89%) responded to the interview and 6,354 reported for physical examinations (12). Altogether 6,354 subjects (79% of the original sample of 8,028 subjects) were included in the analysis of the present study from which PI*Z genotyping was performed in a geographically stratified random subsample of 2,482 subjects. The map of the study localities is found at www.terveys2000.fi/locations.html.

### Genotyping

Genotyping of the alpha-1-antitrypsin gene (*SERPINA1*) was performed at FIMM Technology Centre, University of Helsinki.

Genotyping of the M (Malton) allele and S allele (rs17580) was performed using the Agena MassARRAY system and the iPLEX Gold assays (Agena Bioscience, San Diego, CA, USA). In this method, allele discrimination is based on primer extension with single mass-modified nucleotides followed by MALDI-TOF mass spectrometry. All reactions are designed in multiplexes of up to 35 SNPs, by use of Assay Design v2.0 software (Agena Bioscience). Genotyping reactions were performed on 20 ng of dried genomic DNA in 384-well plate formats according to manufacturer's recommendations. Concentrations of the extension primers were adjusted according to their mass and varied between 7 and 24.6 µM. The data were collected using the MassARRAY Compact System (Agena Bioscience), and the genotypes were called using Typer 4 software (Agena Bioscience). The Z allele (rs28929474) variation was assessed by use of TaqMan SNP Endoint genotyping (Applied Biosystems, Foster City, CA, USA) on the LightCycler 480 system (Hoffman-La Roche Ltd, Basel, Switzerland). A total of 2,496 DNA samples and 25 duplicate samples were run. Eighteen of the samples genotyped had no signal at all or were discarded manually as not reliable due to very low signal intensity. For quality control reasons, the genotype calls on both genotyping systems were checked manually and corrected when necessary. In addition, genotyping quality of all assays was examined by a detailed QC procedure consisting of success rate check, duplicated samples, water controls, and Hardy–Weinberg Equilibrium testing.

**Fig. 1:**
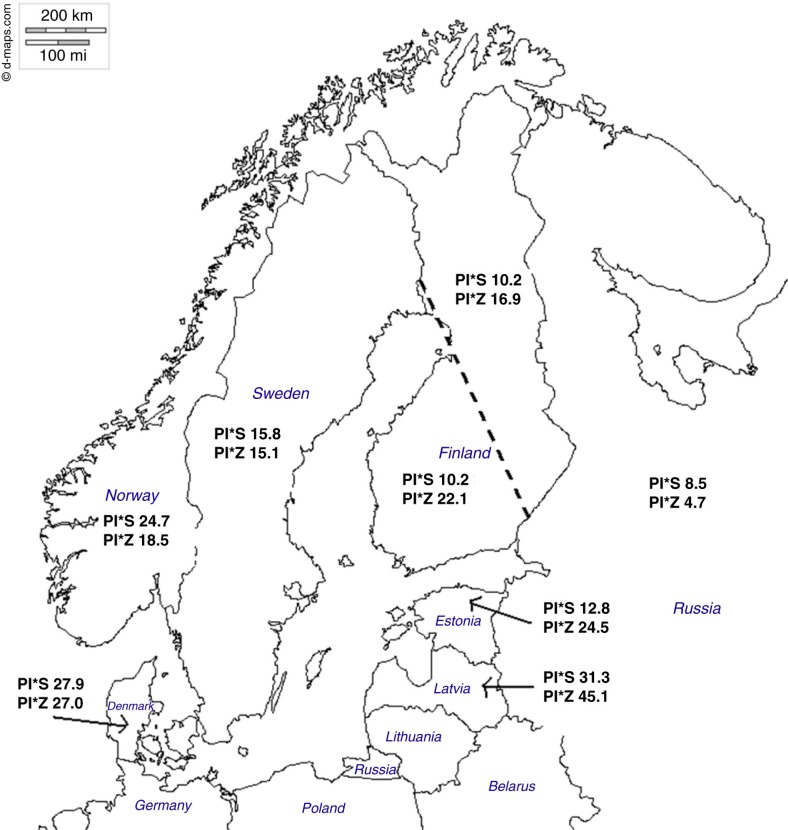
Prevalence of PI*Z and PI*S alleles in Finland and its neighboring countries. Prevalences are expressed as total number of alleles per 1,000 genes of all PI types. Prevalence numbers other than Finland taken from de Serres [de Serres et al. (10)]. The number of studied subjects were 10,096 in Denmark, 4,492 in Norway, 1,062 in Sweden, 1,850 in Estonia, 288 in Latvia, and 2,787 in Russia (numbers taken from Blanco I [Blanco et al. (9)] Table 1). The eastern–western areas of Finland are divided with the dashed black line as described by Kittles et al. (Map taken from www.d-maps.com/).

The eastern–western areas of Finland were divided as described by Kittles et al. (Fig. 1) (11). The population size was 1,241 people in both groups. The statistical power calculations with two-sided tests, *p*-value of 0.05, and the desired power of 0.80 gave a needed sample size of 717 people in both groups. With one-sided test and a *p*-value of 0.01, the needed sample size was calculated to be 917 people in both groups. Allele frequencies are expressed as the total number of Z and S alleles per 1,000 genes of all PI types.

## Results

The observed frequencies of PI*Z, PI*S, and PI*M (Malton) alleles and estimated numbers of corresponding allele carriers in Finnish population are shown in Table 1. None of the study subjects had PI*SS genotype and only one with PIZZ genotype was found in this population. Moreover, no PI*M (Malton) allele was found.

**Table 1:** Allele frequencies and number of carriers in Finnish population. The total population in Finland (year 2000) used in these calculations was 5,181,000 (taken from Statistics Finland year 2000)

	Sample size	Allele frequencies (%)	Allele frequencies (total number of alleles per 1,000 genes of all PI types)	Number of carriers in Finnish population
PI*Z	2,482	3.91	19.7	202,577
PI*S	6,354	2.04	10.2	105,692
PI*M (Malton)	6,354	0.00	0.0	0

In the eastern PI*Z group, which consisted of 1,241 persons, we found 42 carriers, giving the frequency of 42/1,241=3.38% (95% CI=2.51–4,54%), and an allele frequency of 1,000×(42/2,482)=16.9. In the western PI*Z group, which consisted of 1,241 persons, we found 55 carriers, giving the frequency of 55/1,241=4.43% (95% CI=3,42–5,72%), and an allele frequency of 1,000×(55/2,482)=22.1. This difference was not statistically significant (*p*-value for difference=0.17). The results are shown in Fig. 1.

## Discussion

Severe early onset of emphysema caused by the deficiency of alpha-1-antitrypsin was first described in 1963 (13). Soon after this discovery, Fagerholm et al. (8) published the prevalences of serum PI types in some Lappish and Finnish populations. The phenotypes of 468 Lappish persons were determined, and only one Finnish Lapp had the phenotype MZ. Though, the Z allele was practically not found at all in the Lappish population. Moreover, 222 Finns (other than Lappish) were studied, and the frequency of PI*Z was 4.5/1,000 and the PI*S allele was not found at all. In the study by Arnaud et al. (6), alpha-1-antitrypsin phenotypes of 548 normal Finnish persons were determined by isoelectric focusing in polyacrylamide gel. The frequencies obtained were PI*Z 13.7/1,000 and PI*S 17.3/1,000. Furthermore, Arvilommi (7) determined these alleles from a population of 1,037. The population material comprised 1,037 serum samples from healthy blood donors (with no medications) in Turku, a town in western Finland in 1970. The allele frequencies were PI*Z 6.7/1,000 and PI*S 4.8/1,000.

We determined alpha-1-antitrypsin PI*Z and PI*S genotypes from a representative Finnish population sample. The genotyping of the PI*Z allele on Agena MassARRAY turned out to be methodologically difficult. There are other SNPs in the close vicinity of PI*Z that interfere with the SNP of interest and make assay design impossible. The PI*Z allele genotyping was instead performed using TaqMan SNP Endoint genotyping on the LightCycler 480 system. Because of this fact and due to a limited research grant, we had to cut down our sample size regarding the Z allele to 2,496 persons. The subsample was geographically balanced to allow analyses regarding an east–west difference. Despite sample size reduction, the studied population was still significantly bigger than in the earlier papers.

According to our study, the prevalence of PI*S allele in Finland is slightly lower than in Scandinavia where the prevalence is around 20/1,000 (10). The PI*Z prevalence in Scandinavia has been reported to be around 20–30/1,000, as seems to be the case in Finland too. We also analyzed whether there is an east–west difference in these prevalences as the earlier studies suggested. Although PI*Z was slightly more common in the western part of the country, no statistically significant differences between these two regions were found.

In conclusion, we determined the prevalences of alpha-1-deficiency alleles PI*Z, PI*S, and PI*M (Malton) in the Finnish population. The number of carriers of the two most common deficiency alleles (PI*Z and PI*S) is significantly higher than previously estimated. The prevalences are in line with the findings in the neighboring countries, and no clear difference was found between eastern and western parts of the country.
